# Constructal Design of an Arrow-Shaped High Thermal Conductivity Channel in a Square Heat Generation Body

**DOI:** 10.3390/e22040475

**Published:** 2020-04-20

**Authors:** Fengyin Zhang, Huijun Feng, Lingen Chen, Jiang You, Zhihui Xie

**Affiliations:** 1 Institute of Thermal Science and Power Engineering, Wuhan Institute of Technology, Wuhan 430205, China; zfy1125546402@163.com (F.Z.); huijunfeng@139.com (H.F.); jsjdyoujiang@163.com (J.Y.); 15071081343@163.com (Z.X.); 2 School of Mechanical & Electrical Engineering, Wuhan Institute of Technology, Wuhan 430205, China; 3 College of Power Engineering, Naval University of Engineering, Wuhan 430033, China

**Keywords:** constructal theory, arrow-shaped high thermal conductivity channel, maximum temperature difference minimization, multi-degree of freedom optimization, generalized thermodynamic optimization

## Abstract

A heat conduction model with an arrow-shaped high thermal conductivity channel (ASHTCC) in a square heat generation body (SHGB) is established in this paper. By taking the minimum maximum temperature difference (MMTD) as the optimization goal, constructal designs of the ASHTCC are conducted based on single, two, and three degrees of freedom optimizations under the condition of fixed ASHTCC material. The outcomes illustrate that the heat conduction performance (HCP) of the SHGB is better when the structure of the ASHTCC tends to be flat. Increasing the thermal conductivity ratio and area fraction of the ASHTCC material can improve the HCP of the SHGB. In the discussed numerical examples, the MMTD obtained by three degrees of freedom optimization are reduced by 8.42% and 4.40%, respectively, compared with those obtained by single and two degrees of freedom optimizations. Therefore, three degrees of freedom optimization can further improve the HCP of the SHGB. Compared the HCPs of the SHGBs with ASHTCC and the T-shaped one, the MMTD of the former is reduced by 13.0%. Thus, the structure of the ASHTCC is proven to be superior to that of the T-shaped one. The optimization results gained in this paper have reference values for the optimal structure designs for the heat dissipations of various electronic devices.

## 1. Introduction

Nowadays, electronic information technology is developing rapidly. Many new electronic components are widely used in various aspects, such as national defense, industry, science and technology, and social life. At present, arranging the high thermal conductivity channel (HTCC) material is one of the common methods to dissipate the heat of the electronic component.

Bejan [1] stated the constructal law after further studying the formation of urban street networks, and applied it to the optimization of the heat dissipation structure of an electronic device (ED) [2]. Since the introduction of the constructal theory [3,4,5,6,7,8,9,10,11,12,13,14,15,16,17,18,19,20,21,22,23], it has been applied to design various heat dissipation bodies, such as rectangular [24,25,26,27,28,29,30,31], triangular [32,33,34,35,36,37], square [38,39,40,41,42,43,44,45,46,47] and discal [48,49,50,51,52,53,54,55,56,57,58,59] bodies, pin-fins [60,61], fork-shaped fins [62,63], generating heat plate [64,65], radiant enclosures [66,67], and heat storage systems [68] among others.

In the constructal designs of the square bodies, Lorenzini et al. [38] set up a heat conduction model (HCM) with X-shaped HTCC in a square heat generation body (SHGB), optimized the structure of X-shaped HTCC with minimum hot spot temperature (HST), and found that the X-shaped HTCC was evidently superior to the I-shaped one. Lorenzini et al. [38] further established a non-uniform HCM, and showed that the heat conduction performance (HCP) of the new HTCC was about 10% higher than that of the HTCC with uniform one. Hajmohammadi et al. [41,42] built the HCMs with three new HTCCs in the SHGBs, and found that their dimensionless maximum temperature differences (DMTDs) after constructal optimizations were lower than those with X- and I-shaped HTCCs. Feng et al. [43] set up an HCM with “+” shaped HTCC in an SHGB, and reduced the DMTD of the SHGB by 12.11% after the use of optimized HTCC with variable cross-section. Lorenzini et al. [44] established an I-shaped HTCC model in an SHGB, and effectively reduced the HST of the SHGB after constructal design. Konan and Cetkin [45] optimized the construct of a snowflake-shaped HTCC in an SHGB, and found that the optimal construct of the HTCC with minimum DMTD was very close to the shape of a snowflake in nature. Hajmohammadi and Rezaei [46] optimized the distributions of HTCCs with two branches in an SHGB based on a local recursive algorithm. The result displayed that the optimized HST was lower than those derived by most models of the discussed literature. Hajmohammadi et al. [47] built an HCM with multistage irregular dendritic HTCC in an SHGB, and found that the HCP was improved by up to 61% compared with the discussed optimal results in the literatures.

There are many possible geometry shapes of high thermal conductivity channels. The geometry shape of a high thermal conductivity channel has important effects of the heat conduction performance, and a more effective geometry shape is meaningful for engineering practice. Therefore, based on Refs. [38,39,40,41,42,43,44,45,46,47], an HCM with an arrow-shaped HTCC (ASHTCC) in an SHGB will be established in this paper. Aiming at the objective of DMTD, the construct of the ASHTCC in the SHGB will be optimized by using constructal theory. Single, two, and three degrees of freedom optimizations will be introduced and compared. The gained optimization results have reference values for the optimal structure designs for the heat dissipations of various electronic devices.

## 2. Model Establishment

The physical and mathematical models are established as follows, respectively.

### 2.1. Physical Model

**Figure 1:**
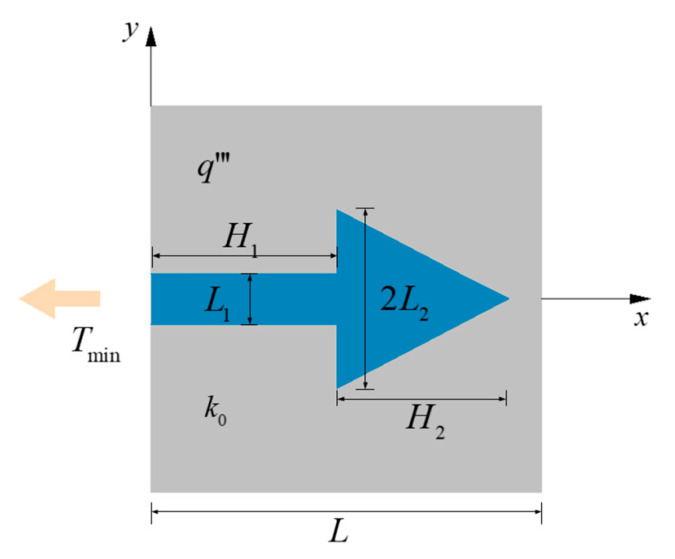
HCM of an ASHTCC in an SHGB.

Figure 1 shows the HCM of an ASHTCC in a square heat generation body. The side length of the SHGB is L. The heat generation rate per unit volume of the SHGB (thermal conductivity $k_{0}$) is $q^{\prime \prime \prime }$. The heat flows into the ASHTCC (thermal conductivity is $k_{p}$), and then flows out of the SHGB from the left side of the ASHTCC (constant temperature $T_{\min}$). The ratio of the thermal conductivities (TCs) is defined as $\tilde{k}=k_{p}/k_{0}$. As shown in Figure 1, the ASHTCC is composed of a triangular arrow tip and rectangular arrow tail, the characteristic sizes of which are $L_{1}$, $L_{2}$, $H_{1}$, and $H_{2}$, respectively. It can be concluded that the temperature of each point in the area of SHGB is higher than that of $T_{\min}$. Except for the constant temperature at the left side of the ASHTCC, the other boundaries of the SHGB are all adiabatic.

The area $A_{0}$ of the ASHTCC can be expressed as: $A_{0}=H_{1}L_{1}+H_{2}L_{2}$. The whole area of the SHGB is $L^{2}$. The area ratio ϕ of the HTCC material to the SHGB is:

(1) $$\phi =\frac{H_{1}L_{1}+H_{2}L_{2}}{L^{2}}={\tilde{H}}_{1}{\tilde{L}}_{1}+{\tilde{H}}_{2}{\tilde{L}}_{2}$$

where $L_{1}$, $L_{2}$, $H_{1}$, and $H_{2}$ are nondimensionalized as: ${\tilde{L}}_{1}=L_{1}/L$, ${\tilde{L}}_{2}=L_{2}/L$, ${\tilde{H}}_{1}=H_{1}/L$, and ${\tilde{H}}_{2}=H_{2}/L$, respectively.

### 2.2. Mathematical Model

Because the model in Figure 1 is symmetric with respect to the *Y*-axis, the temperature distributions will be equal to each other at both sides of the *Y*-axis. Therefore, only half of the geometry (y≥0) will be simulated in the following. It is not a simple one-dimensional HCM due to the ASHTCC, thus a numerical calculation method should be adopted. The two-dimensional dimensionless heat conduction equations in a steady state and with constant thermal conductivity of the low and high TC materials are

(2) $$\frac{\partial^{2}\tilde{T}}{\partial {\tilde{x}}^{2}}+\frac{\partial^{2}\tilde{T}}{\partial {\tilde{y}}^{2}}+1=0$$

(3) $$\tilde{k}\left(\frac{\partial^{2}\tilde{T}}{\partial {\tilde{x}}^{2}}+\frac{\partial^{2}\tilde{T}}{\partial {\tilde{y}}^{2}}\right)=0$$

where $\tilde{T}=(T-T_{\min})/(q^{\prime \prime \prime }L^{2}/k_{0})$, $\tilde{k}=k_{p}/k_{0}$, $\tilde{x}=x/L$, and $\tilde{y}=y/L$. The dimensionless boundary constraints in the y≥0 region are:

(4) $$\tilde{T}=0,\;\tilde{x}=0,\;0\leq \tilde{y}<{\tilde{L}}_{1}/2$$

(5) $$\frac{\partial \tilde{T}}{\partial \tilde{x}}=0\;\left\{ \begin{array}{l} \begin{array}{cc} \tilde{x}=0, & {\tilde{L}}_{1}/2\leq \tilde{y}\leq 1/2 \end{array}\\ \begin{array}{cc} \tilde{x}=1, & 0\leq \tilde{y}\leq 1/2 \end{array} \end{array}\right.$$

(6) $$\frac{\partial \tilde{T}}{\partial \tilde{y}}=0\;\left\{ \begin{array}{l} \begin{array}{cc} \tilde{y}=0, & 0\leq \tilde{x}\leq 1 \end{array}\\ \begin{array}{cc} \tilde{y}=1/2,\; & 0\leq \tilde{x}\leq 1 \end{array} \end{array}\right.$$

where ${\tilde{L}}_{1}=L_{1}/L$.

Assuming that the contact thermal resistance between HTCC and low TC material is negligible, the continuity equation of heat flux between $k_{p}$ and $k_{0}$ materials is

(7) $${\left(\partial T/\partial n\right)}_{k_{0}}=\tilde{k}{\left(\partial T/\partial n\right)}_{k_{p}}$$

The DMTD in the SHGB is defined as

(8) $$\Delta {\tilde{T}}_{1}=(T_{\max}-T_{\min})/(q^{\prime \prime \prime }L^{2}/k_{0})$$

where $T_{\max}$ is the HST in the SHGB.

The finite element software (Comsol Multiphysics) can be used to solve Equations (1)–(7). Combining with Equation (8), the DMTD can be obtained. Under the condition that the area ratio ϕ of the HTCC material in Equation (1) is given, the dimensionless height ${\tilde{H}}_{2}$ of the arrow tip can be expressed by the dimensionless width ${\tilde{L}}_{1}$ of the arrow tail, dimensionless bottom length ${\tilde{L}}_{2}$ of the arrow tip, and dimensionless length ${\tilde{H}}_{1}$ of the arrow tail. Finally, the DMTD is related to the structure parameters ${\tilde{L}}_{1}$, ${\tilde{L}}_{2}$ and ${\tilde{H}}_{1}$ (${\tilde{L}}_{1}=L_{1}/L$, ${\tilde{L}}_{2}=L_{2}/L$ and ${\tilde{H}}_{1}=H_{1}/L$), respectively.

## 3. Constructal Design of the ASHTCC

Three degrees of freedom are considered in the constructal design problem of the ASHTCC. Constructal designs of the ASHTCC based on single, double, and three degrees of freedom optimizations will be successively conducted as follows. The initial parameters are set as follows: The constant temperature $T_{\min}=300\;K$, the heat generation rate $q^{\prime \prime \prime }=500\left(W/m^{3}\right)$, and the low thermal conductivity of materials $k_{0}=2\left(W/m\cdot K\right)$.

### 3.1. Constructal Design Based on Single Degree of Freedom Optimization

For the fixed ${\tilde{L}}_{2}$ and ${\tilde{H}}_{1}$, the DMTD is selected as the performance index, and the constructal design of the ASHTCC is conducted by varying the width ${\tilde{L}}_{1}$, i.e., single degree of freedom optimization (SDFO). The relevant parameters in the calculations are given as follows: the area ratio of the HTCC material is ϕ=0.1, dimensionless bottom length is ${\tilde{L}}_{2}=0.4$, dimensionless height is ${\tilde{H}}_{2}=0.1$, and TC ratio is ${\tilde{k}}_{0}=200$.

**Figure 2:**
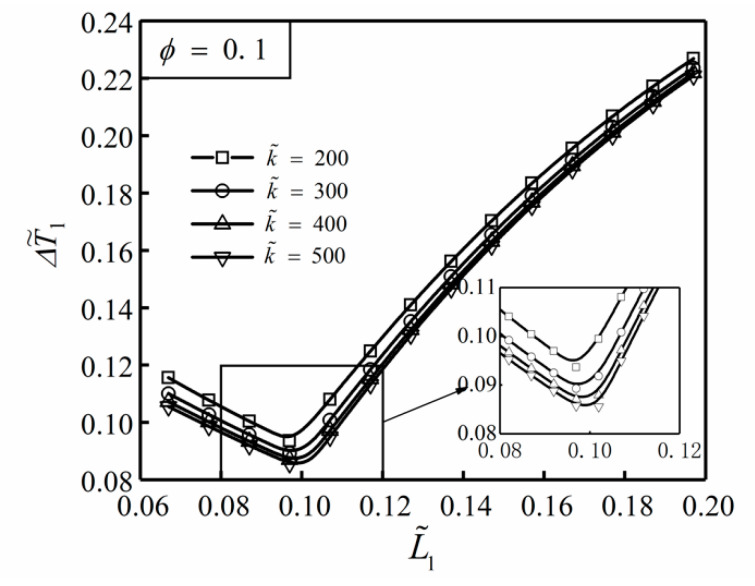
Influence of $\tilde{k}$ on the relationship between $\Delta {\tilde{T}}_{1}$ and ${\tilde{L}}_{1}$ with ϕ=0.1.

Figure 2 shows the influence of the TC ratio $\tilde{k}$ on the relationship between the DMTD $\Delta {\tilde{T}}_{1}$ and dimensionless width ${\tilde{L}}_{1}$ of the ASHTCC with ϕ=0.1. From Figure 2, under the conditions of area ratio ϕ=0.1 and dimensionless arrow tip area ${\tilde{L}}_{2}{\tilde{H}}_{2}=0.04$, the minimum value of $\Delta {\tilde{T}}_{1}$ can be obtained by selecting reasonable ${\tilde{L}}_{1}$. When ${\tilde{L}}_{1}$ is close to ${\tilde{L}}_{1}=0.1$, the HCP of the SHGB is better. In addition, when ${\tilde{L}}_{1}$ remains constant, $\Delta {\tilde{T}}_{1}$ gradually decreases with the increase in $\tilde{k}$. This illustrates that increasing the TC ratio can also improve the HCP of the SHGB.

**Figure 3:**
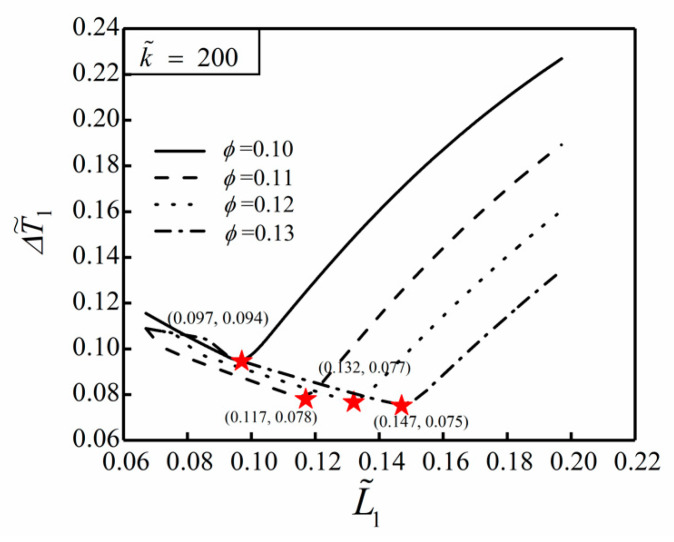
Influence of ϕ on the relationship between $\Delta {\tilde{T}}_{1}$ and ${\tilde{L}}_{1}$ with $\tilde{k}=200$.

**Figure 4:**
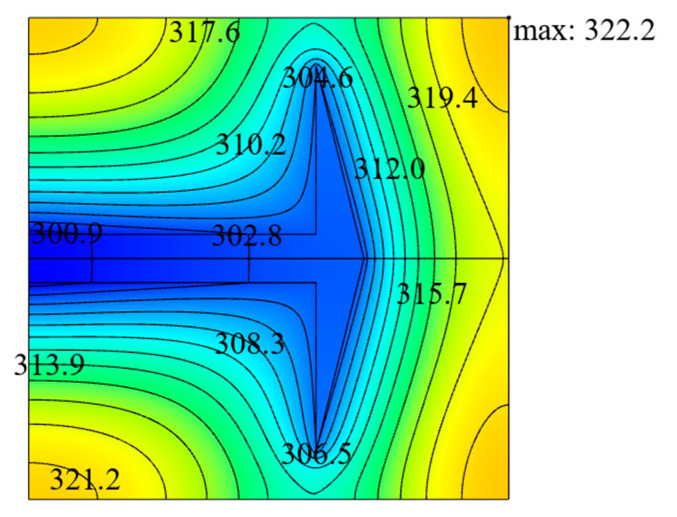
Temperature profile corresponding to the optimal construct based on SHGB.

Figure 3 shows the influence of the area ratio ϕ of HTCC material on the relationship between the DMTD $\Delta {\tilde{T}}_{1}$ and dimensionless width ${\tilde{L}}_{1}$ of the ASHTCC with $\tilde{k}=200$. When ϕ changes in a small range, the heat generation rate of the SHGB will slightly change, which is ignored in the following analyses. From Figure 3, it can be seen that when ϕ increases, the minimum value of the DMTD $\Delta {\tilde{T}}_{1}$ decreases and ${\tilde{L}}_{1,\mathrm{opt}}$ increases gradually. This indicates that the larger the HTCC area is, the better the HCP of the SHGB becomes. For approximately the same heat generation rate of the SHGB, when the area ratio increases from ϕ=0.10 to ϕ=0.13, the minimum DMTD decreases by 20.21%, and the HCP of the SHGB becomes better. When ϕ=0.1 and $\tilde{k}=300$, the minimum DMTD of the SHGB obtained by finite element method is $\Delta {\tilde{T}}_{1,m}=0.088$, and the temperature profile corresponding to the optimal construct is shown in Figure 4.

### 3.2. Constructal Design Based on Two Degrees of Freedom Optimization

The dimensionless bottom length ${\tilde{L}}_{2}$ and dimensionless length ${\tilde{H}}_{1}$ are fixed in Section 3.1, and the constructal design of the ASHTCC will be conducted by varying the width ${\tilde{L}}_{1}$ and bottom length ${\tilde{L}}_{2}$ simultaneously in this section. In the two degrees of freedom optimization (TWDFO), the arrow tip area remains unchanged at ${\tilde{L}}_{2}{\tilde{H}}_{2}=0.04$.

**Figure 5:**
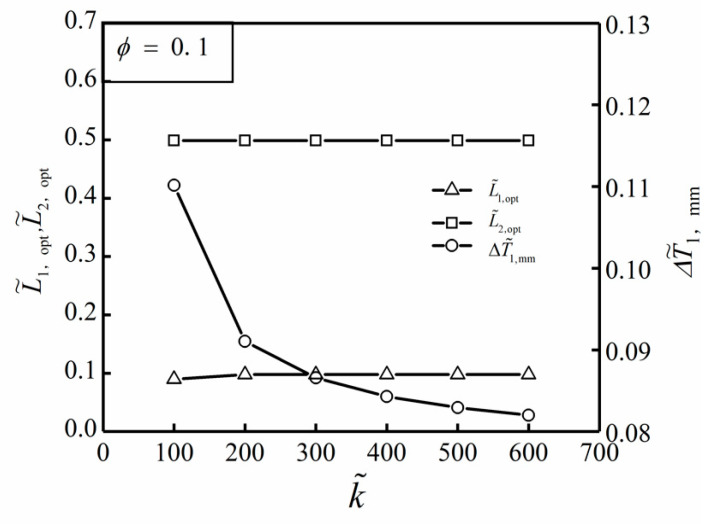
Influences of $\tilde{k}$ on the optimal results of TWDFO with ϕ=0.1.

**Figure 6:**
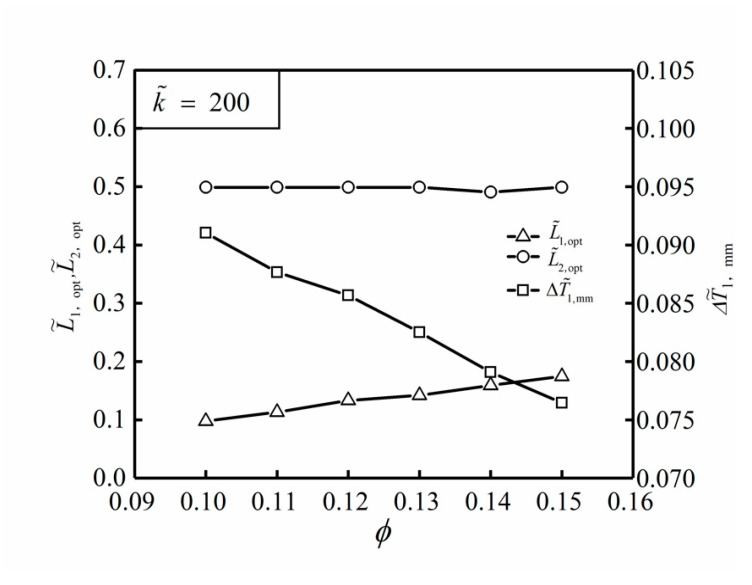
Influences of ϕ on the optimal results of TWDFO with $\tilde{k}=200$.

**Figure 7:**
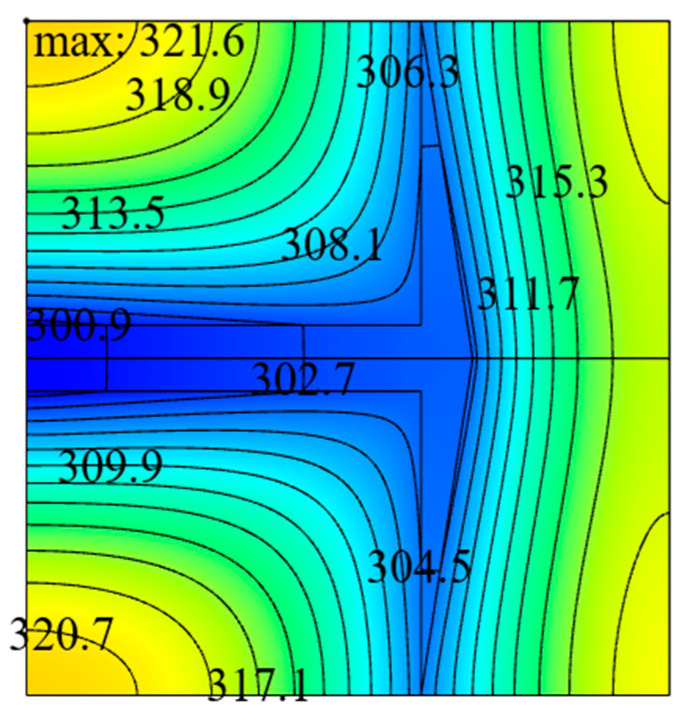
Temperature profile corresponding to the optimal construct based on TWDFO with $\tilde{k}=300$ and ϕ=0.1.

Figure 5 shows the influences of TC ratio $\tilde{k}$ on the optimal results ($\Delta {\tilde{T}}_{1,\mathrm{mm}}$, ${\tilde{L}}_{1,\mathrm{opt}}$ and ${\tilde{L}}_{2,\mathrm{opt}}$) of TWDFO with ϕ=0.1. From Figure 5, it can be seen that the optimal construct of the ASHTCC after TWDFO is ${\tilde{L}}_{1,\mathrm{opt}}\approx 0.1$ and ${\tilde{L}}_{2,\mathrm{opt}}\approx 0.499$. In this case, the HTCC shape becomes flat. When the TC ratio $\tilde{k}$ increases, the changes of ${\tilde{L}}_{1,\mathrm{opt}}$ and ${\tilde{L}}_{2,\mathrm{opt}}$ are not obvious, while the double minimum DMTD $\Delta {\tilde{T}}_{1,\mathrm{mm}}$ decreases gradually. Figure 6 further shows the influences of the area ratio ϕ of HTCC material on the optimal results ($\Delta {\tilde{T}}_{1,\mathrm{mm}}$, ${\tilde{L}}_{1,\mathrm{opt}}$ and ${\tilde{L}}_{2,\mathrm{opt}}$) of TWDFO with $\tilde{k}=200$. According to Figure 6, when the area ratio ϕ increases, the change of ${\tilde{L}}_{2,\mathrm{opt}}$ is not obvious, and ${\tilde{L}}_{1,\mathrm{opt}}$ increases gradually, however $\Delta {\tilde{T}}_{1,\mathrm{mm}}$ decreases gradually. When ϕ=0.1 and $\tilde{k}=300$, the double minimum DMTD of the SHGB obtained by finite element method is $\Delta {\tilde{T}}_{1,\mathrm{mm}}=0.086$, and the temperature profile corresponding to the optimal construct is shown in Figure 7.

### 3.3. Constructal Design Based on Three Degrees of Freedom Optimization

The dimensionless length ${\tilde{H}}_{1}$ is fixed in Section 3.2, and the constructal design of the ASHTCC will be further conducted by varying the width ${\tilde{L}}_{1}$, bottom length ${\tilde{L}}_{2}$, and dimensionless length ${\tilde{H}}_{1}$ simultaneously in this section, i.e., the three degrees of freedom optimization (THDFO).

**Figure 8:**
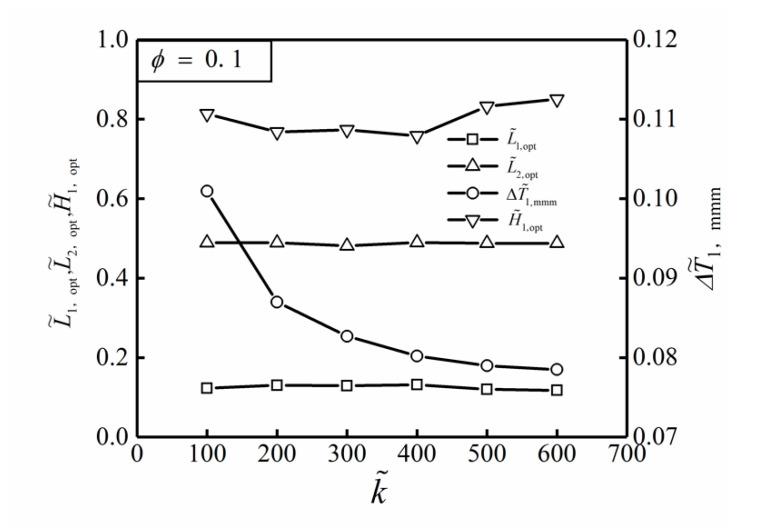
Influences of $\tilde{k}$ on the optimal results of THDFO with ϕ=0.1.

Figure 8 shows the influences of the TC ratio $\tilde{k}$ on the optimal results ($\Delta {\tilde{T}}_{1,\mathrm{mmm}}$, ${\tilde{L}}_{1,\mathrm{opt}}$, ${\tilde{L}}_{2,\mathrm{opt}}$ and ${\tilde{H}}_{1,\mathrm{opt}}$) of THDFO with ϕ=0.1. From Figure 8, it can be seen that the optimal construct of the ASHTCC after THDFO is ${\tilde{L}}_{1,\mathrm{opt}}=0.197$, ${\tilde{L}}_{2,\mathrm{opt}}=0.488$ and ${\tilde{H}}_{1,\mathrm{opt}}=0.645$. This illustrates that the HCP of the SHGB is still better when the shape of the HTCC tends to be flat. When $\tilde{k}$ increases, the changes of ${\tilde{L}}_{1,\mathrm{opt}}$, ${\tilde{L}}_{2,\mathrm{opt}}$, and ${\tilde{H}}_{1,\mathrm{opt}}$ are not obvious, while the triple minimum DMTD $\Delta {\tilde{T}}_{1,\mathrm{mmm}}$ decreases gradually. When the TC ratio increases from $\tilde{k}=100$ to $\tilde{k}=600$, the triple minimum DMTD decreases from $\Delta {\tilde{T}}_{1,\mathrm{mmm}}=0.101$ to $\Delta {\tilde{T}}_{1,\mathrm{mmm}}=0.079$. $\Delta {\tilde{T}}_{1,\mathrm{mmm}}$ is decreased by 21.8%, and the HCP of the SHGB is significantly improved.

**Figure 9:**
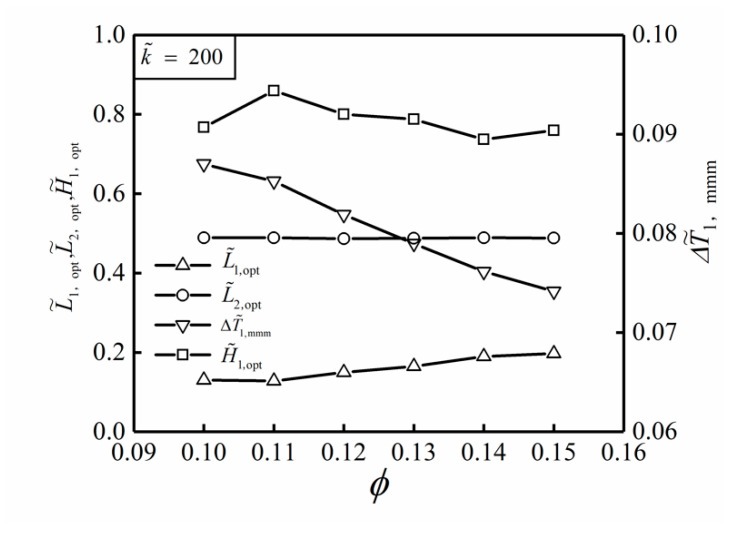
Influences of ϕ on the optimal results of THDFO with $\tilde{k}=200$.

Figure 9 shows the influences of the area ratio ϕ on the optimal results ($\Delta {\tilde{T}}_{1,\mathrm{mmm}}$, ${\tilde{L}}_{1,\mathrm{opt}}$, ${\tilde{L}}_{2,\mathrm{opt}}$ and ${\tilde{H}}_{1,\mathrm{opt}}$) of THDFO with ϕ=0.1. From Figure 9, when the area ratio ϕ increases, the change of ${\tilde{L}}_{2,\mathrm{opt}}$ is not obvious, ${\tilde{L}}_{1,\mathrm{opt}}$ increases gradually, and $\Delta {\tilde{T}}_{1,\mathrm{mmm}}$ decreases gradually. When the area ratio increases from ϕ=0.10 to ϕ=0.15, the triple minimum DMTD decreases from $\Delta {\tilde{T}}_{1,\mathrm{mmm}}=0.087$ to $\Delta {\tilde{T}}_{1,\mathrm{mmm}}=0.074$. $\Delta {\tilde{T}}_{1,\mathrm{mmm}}$ is decreased by 14.9%, and the HCP of the SHGB is significantly improved.

### 3.4. Performance Comparison for Different Degrees of Freedom

**Figure 10:**
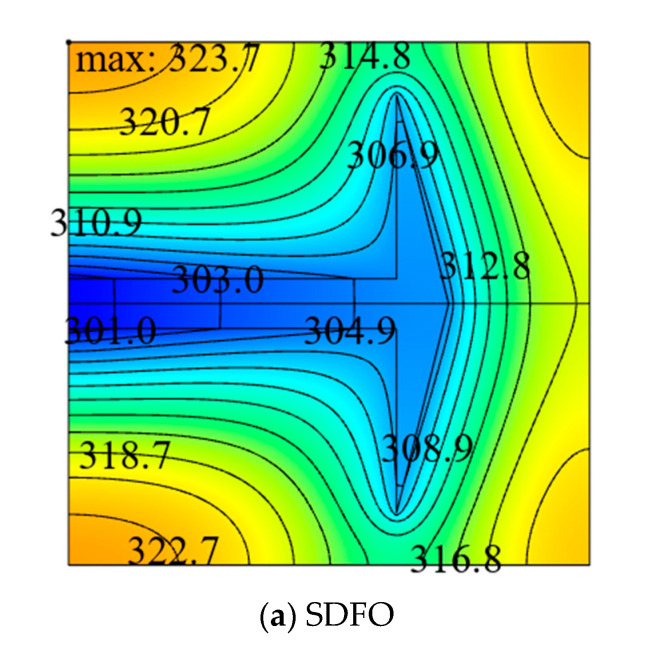

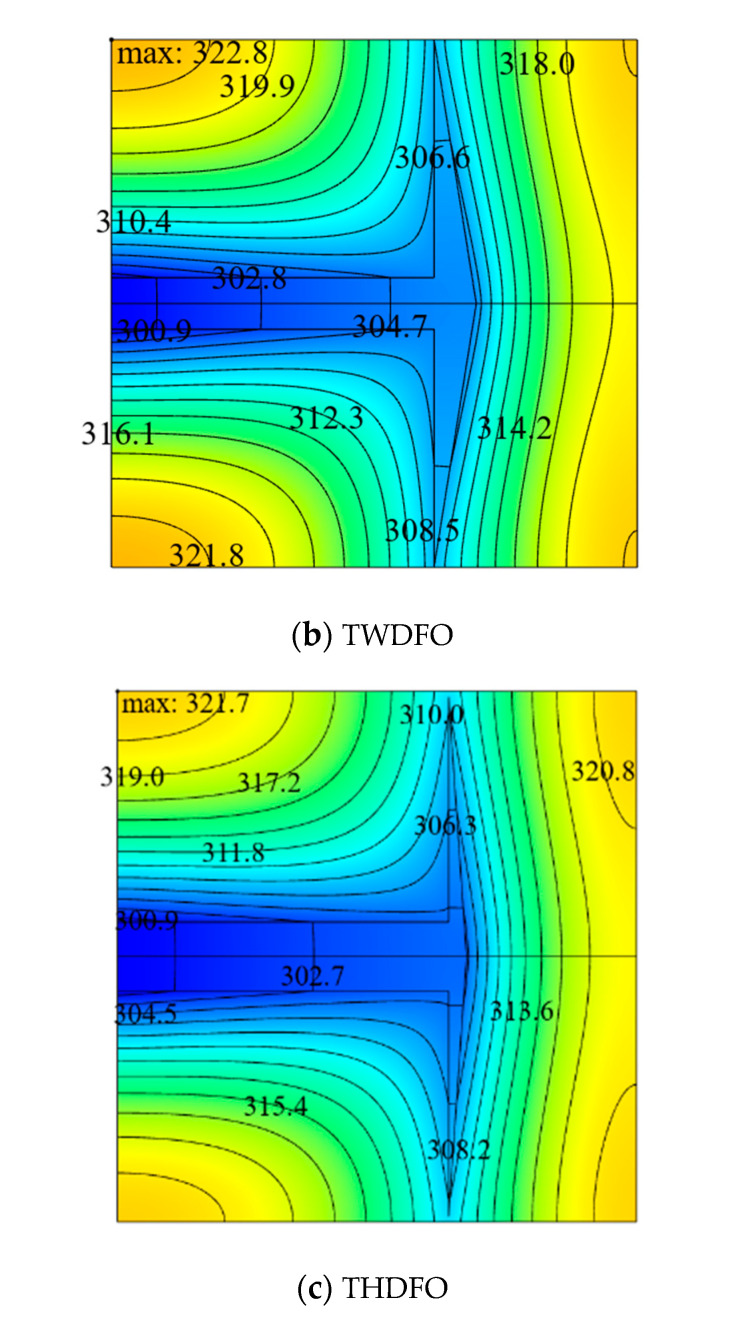
Temperature profiles corresponding to the optimal constructs obtained by single, double and three degrees of freedom optimizations with $\tilde{k}=200$ and ϕ=0.1.

Under the conditions of ϕ=0.1 and $\tilde{k}=200$, the optimal constructs and corresponding temperature profiles of the ASHTCC obtained by single, double, and three degrees of freedom optimizations are shown in Figure 10, respectively. From the temperature profiles, it can be seen that the HSTs in the SHGBs are 323.7 K, 322.8 K, and 321.7 K, and the corresponding DMTDs are 0.095, 0.091, and 0.087, respectively. The DMTD of the SHGB after THDFO is respectively reduced by 4.40% and 8.42% compared with those after TWDFO and SDFO. It can be seen that the optimal structure of the ASHTCC obtained by THDFO significantly reduces the HST and improves the HCP of the SHGB.

## 4. Performance Comparison of the Square Heat Generation Bodies with Arrow- and T-Shaped High Thermal Conductivity Channels

**Figure 11:**
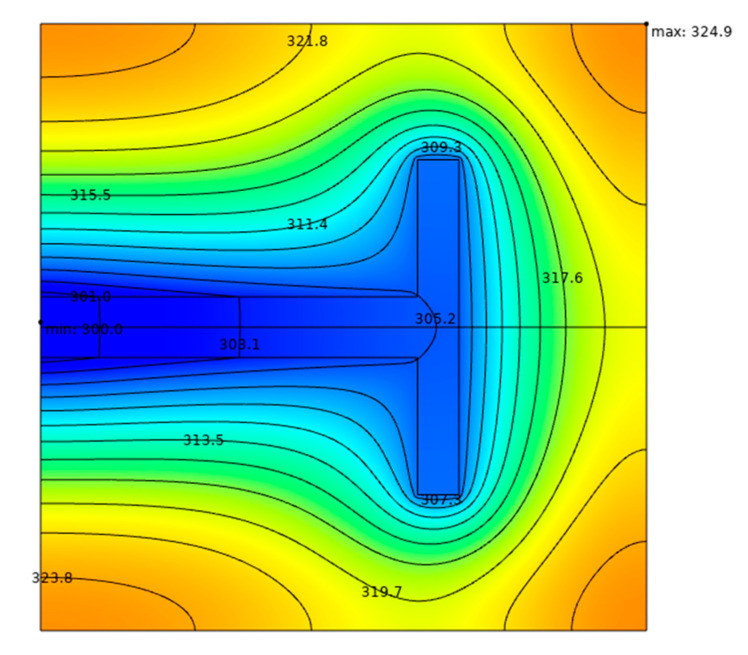
Optimal temperature profile of the SHGB with T-shaped HTCC, $\tilde{k}=200$ and ϕ=0.1.

To compare the HCP of the square heat generation body with ASHTCC to those with the other HTCCs, the optimal temperature profile of the SHGB with T-shaped HTCC is shown in Figure 11 as an example. From Figure 11, it can be seen that the triple minimum DMTD of the SHGB with T-shaped HTCC is $\Delta {\tilde{T}}_{1,\mathrm{mmm}}=0.100$. Compared the HCP of the SHGB in Figure 10c with that in Figure 11, the DMTD of the former is reduced by 13.0%. Therefore, the ASHTCC exhibits better HCP than the T-shaped HTCC.

## 5. Conclusions

A heat conduction model with ASHTCC in a square heat generation body is built in this paper. Constructal designs of the ASHTCC are conducted based on single, two, and three degrees of freedom optimizations with the objective of minimum DMTD. Optimal constructs of the ASHTCC and optimal temperature profiles are gained. The results are summarized as follows:

(1) For the SDFO, under the conditions of area ratio ϕ=0.1 and dimensionless arrow tip area ${\tilde{L}}_{2}{\tilde{H}}_{2}=0.04$, when ${\tilde{L}}_{1}$ is close to ${\tilde{L}}_{1}=0.1$, the HCP of the SHGB is better. When the area ratio increases from ϕ=0.10 to ϕ=0.13, the minimum DMTD decreases by 20.21%, and the HCP of the SHGB becomes better.

(2) For the TWDFO, the optimal construct of the ASHTCC is ${\tilde{L}}_{1,\mathrm{opt}}\approx 0.1$ and ${\tilde{L}}_{2,\mathrm{opt}}\approx 0.499$. In this case, the shape of the HTCC becomes flat. When the TC ratio $\tilde{k}$ increases, the changes of ${\tilde{L}}_{1,\mathrm{opt}}$ and ${\tilde{L}}_{2,\mathrm{opt}}$ are not obvious, while the double minimum DMTD $\Delta {\tilde{T}}_{1,\mathrm{mm}}$ decreases gradually. When the area ratio ϕ increases, the change of ${\tilde{L}}_{2,\mathrm{opt}}$ is not obvious, and ${\tilde{L}}_{1,\mathrm{opt}}$ increases gradually, however $\Delta {\tilde{T}}_{1,\mathrm{mm}}$ decreases gradually.

(3) For the THDFO, the optimal construct of the ASHTCC is ${\tilde{L}}_{1,\mathrm{opt}}=0.197$, ${\tilde{L}}_{2,\mathrm{opt}}=0.488$ and ${\tilde{H}}_{1,\mathrm{opt}}=0.645$. This illustrates that the HCP of the SHGB is still better when the shape of the HTCC tends to be flat. The DMTD of the SHGB after THDFO is respectively reduced by 4.40% and 8.42% compared with those after TWDFO and SDFO.

(4) Comparing the HCP of the SHGB in Figure 10c with that in Figure 11, the DMTD of the former is reduced by 13.0%. Therefore, the ASHTCC exhibits better HCP than the T-shaped HTCC.

In this paper, the construct of an elemental ASHTCC in the SHGB is optimized. The first-order ASHTCC can be composed of several elemental ASHTCCs. At the same time, the arc structure can be used to replace the cusp structure at the arrow tip. The constructal optimizations of the HTCCs in the SHGB will be further conducted in our future studies by considering the additional model improvements, and better HCPs of the electronic devices will be obtained. Moreover, other optimization objectives, such as entropy generation minimization [69,70,71,72,73,74,75], will also be conducted.

## Abbreviations

ASHTCC	Arrow-shaped high thermal conductivity channel
DMTD	Dimensionless maximum temperature difference
HCM	Heat conduction model
HCP	Heat conduction performance
HST	Hot spot temperature
HTCC	High thermal conductivity channel
SDFO	Single degree of freedom optimization
SHGB	Square heat generation body
TC	Thermal conductivity
TWDFO	Two degrees of freedom optimization
THDFO	Three degrees of freedom optimization
**Nomenclature**	
$A_{0}$	Area, $m^{2}$
k	Thermal conductivity, W/m/K
$\tilde{k}$	Ratio of the thermal conductivity
L	Side length of the SHGB, m
$L_{1}$, $L_{2}$, $H_{1}$, $H_{2}$	Characteristic sizes of the ASHTCC, m
${\tilde{L}}_{1}$, ${\tilde{L}}_{2}$, ${\tilde{H}}_{1}$, ${\tilde{H}}_{2}$	Nondimensional characteristic sizes of the ASHTCC
T	Temperature, K
$q^{\prime \prime \prime }$	Heat generation rate in the SHGB, $W/m^{3}$
ϕ	Area ratio of the HTCC
